# Beyond the Lactate Paradox: How Lactate and Acidity Impact T Cell Therapies against Cancer

**DOI:** 10.3390/antib10030025

**Published:** 2021-06-28

**Authors:** Violet Y. Tu, Asma Ayari, Roddy S. O’Connor

**Affiliations:** 1 Center for Cellular Immunotherapies, Perelman School of Medicine of the University of Pennsylvania, Philadelphia, PA 19104, USA; yitaotu@sas.upenn.edu; 2 Department of Biological Physics, University of Pennsylvania, Philadelphia, PA 19104, USA; 3 Nucleus Biologics, LLC., San Diego, CA 92127, USA; aayari@nucleusbiologics.com; 4 Department of Pathology and Laboratory Medicine, Perelman School of Medicine of the University of Pennsylvania, Philadelphia, PA 19104, USA

**Keywords:** lactic acid, lactate, acidosis, acidic, TME, CAR T-cells, LDHA, VISTA

## Abstract

T cell therapies, including CAR T cells, have proven more effective in hematologic malignancies than solid tumors, where the local metabolic environment is distinctly immunosuppressive. In particular, the acidic and hypoxic features of the tumor microenvironment (TME) present a unique challenge for T cells. Local metabolism is an important consideration for activated T cells as they undergo bursts of migration, proliferation and differentiation in hostile soil. Tumor cells and activated T cells both produce lactic acid at high rates. The role of lactic acid in T cell biology is complex, as lactate is an often-neglected carbon source that can fuel TCA anaplerosis. Circulating lactate is also an important means to regulate redox balance. In hypoxic tumors, lactate is immune-suppressive. Here, we discuss how intrinsic- (T cells) as well as extrinsic (tumor cells and micro-environmental)-derived metabolic factors, including lactate, suppress the ability of antigen-specific T cells to eradicate tumors. Finally, we introduce recent discoveries that target the TME in order to potentiate T cell-based therapies against cancer.

## 1. Introduction

The tumor microenvironment (TME) consists of driver tumor cells, along with a heterogeneous array of other cell types, including fibroblasts, T cells, macrophages, myeloid cells, adipocytes and endothelial cells. These cells are embedded in a matrix that undergoes dynamic remodeling during tumorigenesis [1]. As these diverse cells undergo proliferation and differentiation, their metabolic activities create a distinct extracellular milieu. This can hinder the efficacy of the endogenous sentinels-T cells on patrol for neo-antigens.

In 1889, Dr. Stephen Paget developed the “seed and soil” theory to explain how the TME supports tumorigenesis in breast cancer. In this analogy, the TME (soil) provides a rich niche to support the development and growth of tumor cells. As the TME adapts to support cancer progression, it becomes an increasingly important determinant of outcome and disease severity [2,3]. A number of follow-up studies supported Paget’s theory in several tumor lineages. The use of orthotopic models with tumor-derived cell lines, and in some cases patient cell xenografts provided conclusive evidence that the growth of cancer cells is supported by specific microenvironmental factors found in localised niches [4,5]. Complementary studies implicated the TME in the development of resistance to standard therapies, including anti-tumor immune responses [6]. These initial findings triggered a series of studies to delineate the composition of the TME, and understand how micro-environmental factors suppress T cell function. Such studies unearthed an unequivocal connection between the TME and local cell metabolism.

## 2. Dichotomous Roles for Lactate: Important Energy Source Versus Oncometabolite

### 2.1. Lactate Replenishes NAD^+^ for Glycolysis

Tumor cells, like all cells in the human body, require ATP to support their growth. Consistent with its role as the universal energy source, glucose metabolism supports the biogenesis, progression and evolution of cancer [7]. Glucose crosses the cell surface by facilitative transport, which is a saturable process. Regardless of ambient oxygen levels, tumor cells metabolise glucose by “aerobic” glycolysis. This process involves a series of enzyme-catalysed reactions that generate precursors for macromolecular biosynthesis, ATP to support the energy demands of proliferation, and cofactors to regulate redox balance [8]. The end-product of glycolysis, pyruvate, has alternate fates. After entering the mitochondria, pyruvate can be completely oxidised to carbon dioxide and water in a process known as oxidative phosphorylation. This process results in 36 molecules of ATP. Alternatively, pyruvate can be reduced to lactic acid in the cytoplasm in a reaction catalysed by Lactate dehydrogenase (LDH). Restricting glucose metabolism to the cytoplasm yields a net gain of two molecules of ATP per glucose molecule. There are several reasons why tumor cells may switch to an energy pathway with less inherent capacity to support ATP replenishment. The final step of glycolysis (an LDH-catalysed reduction of pyruvate to lactate) facilitates the intracellular replenishment of NAD^+^, which is an important co-factor supporting redox balance; and co-substrate for anabolic reactions underlying proliferation and differentiation. The oxygen “cost” or requirements to metabolise glucose by oxidative phosphorylation are obviously higher, and this critical metabolite may be limiting in some tumors. Distinguishing between bioenergetic capacity and efficiency is an important principle that is often misunderstood. As glycolysis supports higher rates of ATP production, it is a more efficient means to support the energy demands of clonal expansion and migration.

The biology of lactate metabolism is highly complex, and its immune-suppressive attributes may be limited to hypoxic conditions. As a fuel, lactate oxidation supports ATP replenishment in neurons [9], skeletal myofibers [10] and some tumor cells [11]. Lactate has important roles in metabolism beyond energy production. Circulating lactate provides an important mechanism to regulate redox balance in the kidney, liver, spleen and pancreas [12]. In the Crypts of Lieberkuhn, lactate supports the clonal expansion and differentiation of LGR5+ stem cells [13]. A recent study showed that lactate supports gluconeogenesis and tricarboxylic acid (TCA) cycle function in regulatory T cells, a unique lymphocyte subset with distinct oxidative attributes [14]. Lactate supports TCA cycle anaplerosis in effector T cells [15]. Embedded within this paradox is an important consequence of lactic acid production and secretion: acidification of the extracellular milieu. How this feature of lactate biology impacts T cell function is an active area of research and increasing evidence suggests its immune-suppressive attributes may be limited to hypoxic conditions.

### 2.2. Lactic Acid as an Oncometabolite

Otto Warburg proposed that cancer cells rely on glycolysis rather than oxidative phosphorylation due to intrinsic impairments in their mitochondrial function [16]. Indeed, cancer cells do rely on glycolysis to channel intermediates into anabolic reactions supporting nucleotide, as well as amino acid biosynthesis. However, the theory that cancer cells have dysfunctional mitochondria was later disproven. Oxidative phosphorylation is upregulated in several cancers, including leukemia, lymphoma and pancreatic ductal adenocarcinoma [17]. Tumor cells rely on oxidative phosphorylation for several reasons. The glycolytic enzyme Hexokinase is localised to mitochondrial membranes, and relies on ATP produced via oxidative phosphorylation to initiate glycolysis. Mitochondrial metabolism also supports the anaplerotic replenishment of citrate in the tricarboxylic acid (TCA) cycle.

Heightened tumor cell metabolism and dysregulated perfusion create a unique metabolic milieu in tumors. As glucose levels diminish, interstitial lactate levels rise [18]. Lactic acid accumulation is often viewed as a metabolic checkpoint that provides a barrier to adoptive immunotherapies. Lactate production negatively correlates with T cell infiltration and overall response to T cell immunotherapies in melanoma and lung cancer [19]; additionally, lactate accumulation has been associated with poor outcome, metastasis, and relapse in cervical cancer [20]. Mechanistically, lactate production is catalysed by LDH. Several isoforms of LDH exist. LDHA and LDHB are often described as cytosolic forms, contributing to NAD^+^ replenishment during glycolysis. In contrast, LDHD is localised to the mitochondria. Interestingly, nuclear LDHA translocation has been observed in cervical cancer cells. In the nucleus, LDHA has a promiscuous role, and its specificity is not limited to oxidation-reduction reactions involving lactate and pyruvate. Noncanonical LDHA activation catalyses α-hydroxybutyrate (α-HB) production in the nucleus. LDHA promoted H3K79 hypermethylation at the loci of several antioxidant genes, including SOD1 and CAT, as well as genes involved in Wnt signaling [21]. Dysregulated LDHA levels have been implicated in the progression several cancers including pancreatic, gastric, bladder and endometrial [22]. LDHB expression levels in oral squamous cell carcinoma correlated with poor treatment outcome and response to neoadjuvant chemotherapy [22]. The expression profile of LDH, combined with its intrinsic catalytic activity relative to PDH, as well as the abundance of MCT proteins in tumor cells, facilitate the production, secretion and accumulation of extracellular lactate. How this end-product of glucose metabolism impacts T cell function is an important focus of our discussion below.

Colegio et al., identified a novel signaling role for lactate, independent of acidity, in the tumor environment [23]. Beyond its role in energy metabolism, lactate provides an important signal to bone marrow-derived macrophages undergoing differentiation in solid tumors. They showed that activation of the lactate/HIF-1α/VEGF signaling axis promotes neovascularisation in hypoxic tumors. Lactate induced a broad array of other genes, including ARG1, Fizz1, Mgl1 and Mgl2; collectively, this cohort supports the polarisation of macrophages to an M2 tumor-promoting state.

Cross talk between metabolic pathways and epigenetic reprogramming via the “histone code” is an important aspect of gene regulation in mammalian cells. During this process, the tail ends of core histone proteins are post-translationally modified with functional groups that are replenished by metabolic intermediates and cofactors. Epigenetic remodeling can influence chromatin accessibility, and ultimately, transcriptional status. A number of acyl-CoA metabolites, including acetyl-CoA, propionyl-CoA and succinyl-CoA, can actively mark transcribing regions at promoters and enhancer sites within various genes. The nature and consequence of histone posttranslational modifications are highly specific: H3K27 acetylation within intronic regions is a hallmark of exhausted T cells [24]; higher levels of acetylation at the IFN-γ promoter distinguish memory and effector T cells from their naïve counterparts [25]. Histone lactylation has been recently added to this growing list of epigenetic posttranslational modifications, revealing an important link between lactate biology and gene regulation. 28 lactylation sites on core histone proteins have been identified to date. It was recently shown that lysine lactylation supported ARG1 expression during later stages of M1 macrophage polarisation following LPS stimulation [26]. Epigenetic gene regulation in pancreatic ductal adenocarcinoma is also influenced by lactate metabolism [27]. Given the potential of small molecules targeting lactate production (inhibiting LDH) or secretion (inhibiting MCT) in cancer treatment, future studies are needed to understand their impact on epigenetic remodeling on other immune cells, especially T cells, within the TME.

### 2.3. Challenges in Measuring Local Metabolic Turnover in Tumors

With the renewed interest in understanding how metabolism regulates T cell function, more sensitive approaches to measure local metabolic effects in tumors are being developed. Using intra-tumoral microdialysis, Roslin and colleagues established baseline measurements of several metabolites, including glucose and lactate, in high-grade astrocytomas. Positioning catheters in the belly of the tumor revealed increased glucose consumption relative to peri-tumoral regions; an effect exemplifying the use of glucose as a substrate to fuel the energy demands of tumor growth. Reciprocal increases in lactate were measured peri-tumorally providing further evidence that tumor cell glucose metabolism culminates in lactic acid production and secretion [28]. Similar findings were demonstrated in malignant gliomas and squamous cell carcinomas [29,30]. These shared metabolic features across a number of tumors suggest that high rates of glucose consumption and lactate secretion are evolutionarily beneficial to cancer cell survival [31].

## 3. Metabolic Consequences of Lactic Acid on T Cell Function

### 3.1. T Cell-Therapies against Cancer Are Gaining Widespread Approval

Specificity is an important aspect of targeted approaches against cancer; sparing healthy cells which would otherwise be vulnerable to standard-of-care approaches such as chemotherapy. There has also been a movement towards the use of cell -based therapies to selectively target and eradicate tumor cells. This led to the resurgence of T cells as “living drugs” against cancer. Several iterations of this theme have culminated in the use of genetically engineered receptors (CARs) to redirect T cell specificity in an MHC-independent manner, the use of transgenic T cell receptors (TCR) to recognise distinct neo-peptides, and the use checkpoint blockade to re-invigorate hypofunctional T cells against cancer. Following tumor eradication, a subset of cytolytic effector T cells transition to memory T cells, providing long-lasting immune surveillance. The potential of CAR T-cell therapies to provide durable remissions led to three successive US Food and Drug Administration (FDA) approvals, including Kymriah™ (Tisagenlecleucel) for pediatric patients and young adults with relapsed or refractory B-cell acute lymphoblastic leukemia (R/R B-ALL), Yescarta™ (Axicabtagene ciloleucel) and Breyanzi™ (lisocabtagene maraleucel) for patients with diffuse large B-cell lymphoma (DLBCL) and Tecartus™ (Brexucabtagene autoleucel) for adults with mantle cell lymphoma (MCL) [32,33].

In contrast to their unprecedented impact in hematologic malignancies, the efficacy of antigen-specific T cells in solid tumors has been limited, in part due to the complex metabolic nature of the TME. [34]. Reduced glucose availability impedes the efficacy of adoptively transferred T cells. In murine models of melanoma, extracellular glucose levels can reach a critical threshold that inhibits intracellular glycolytic flux. Decreased levels of the glycolytic intermediate phosphoenolpyruvate (PEP) have multiple consequences on T cell function, including decreased NFAT-mediated transcription, reduced T cell activation and impaired anti-tumor immunity [18].

### 3.2. Contexts Where Acidity Is an Important Checkpoint Limiting T Cell Activation

In 2019, Wu and colleagues identified an important physiologic context where acidosis protects T cell clonal integrity through inhibitory mechanisms. Much like tumor environments, pro-inflammatory states are characterised by increasing acidosis, lactate accumulation, and in some instances hypoxia [35]. Using a Seahorse extracellular flux analyser, Wu et al. quantified the extracellular acidification rate (ECAR), proton production rate (PPR) and glycolytic profile in activated T cells [36]. The group altered the media formulation to adjust intracellular (pHi) and extracellular pH (pHe), and observed a coupling action between the two metrics. In general, proton flux is regulated by monocarboxylate co-transporters (MCTs). The MCT family regulates carbohydrate, amino acid and fatty acid metabolism by catalysing the proton-linked transport of monocarboxylates, including pyruvate, lactate and ketone bodies, across the plasma membrane [36]. In particular, MCT1 and MCT4 prevent intracellular acidification by co-exporting lactate and hydrogen ions across the cell surface. This paper showed that MCT1 and MCT4 are dynamically regulated, highly active at physiologic pH levels and inhibited by ambient acidity. When MCT isoforms are inactivated, H^+^ ions and lactate accumulate intracellularly and impair glycolysis by end-point inhibition. These findings have important implications for understanding how T cell activation is tempered by the acidic state of in the lymph node. Maintaining an acidic rather an alkaline state raises the threshold at which inflammatory cascades could potentially trigger an autoimmune response [36].

Metabolic conditions specific to lymph nodes—particularly their location within adipose depots—may support the homeostatic replenishment of memory T cells [37]. The physiologic context of T cell activation may be an important determinant of overall efficacy. In contrast to T cells activated through their endogenous TCR in lymph nodes, T cells genetically engineered with CARs undergo constitutive activation in hostile tumor environments where low pH is inherently immunosuppressive.

### 3.3. Lactate Versus Acidity: Identifying the Bonafide Barrier to T Cell Function

Relapse following allogeneic hematopoietic cell transplantation (HCT) is often attributed to a progressive loss of function in donor T cells, culminating in diminishing graft versus leukemia response. In a recent study, Uhl and colleagues provided insight into the mechanisms underlying relapse following allogeneic HCT in AML [38]. In the context of “allogeneic immune pressure”, leukemic cells evade T cell lysis by creating a hostile microenvironment enriched with immune suppressive metabolites. Metabolomic screening revealed lactic acid as a top candidate in conditioned media derived from cultured AML cells. Complementary analyses showed elevated lactic acid levels in the plasma of patients following a relapse. Using CFSE dilution assays, they showed that leukemia-derived lactic acid impeded T cell proliferation in vitro. The inhibitory effects of H^+^La^−^ were reversed by conditioning T cells with sodium bicarbonate. Similarly, cytokine production could be rescued by including sodium bicarbonate as a neutralising buffer. As increasing lactic acid levels have been implicated in cancer, it is important to consider which component, when dissociated (H^+^ or La^−^), is the errant factor [39]. Interestingly, the immune-suppressive of lactic acid effects were not attributed to acidity alone, as T cells tolerated a disrupted pH balance (established by the addition of HCL) in the absence of lactate. Using ^13^C_3_ lactic acid and LC/MS approaches, lactic acid is consumed by proliferating T cells, and is integrated into the TCA cycle where it supports the anaplerotic replenishment of several intermediates, including citrate, succinate, fumarate and malate. These data are interesting, as they suggest that there is no bottleneck on lactate accumulation in activated T cells. As the pKa of lactic acid is 3.8, it likely dissociates into lactate anion and hydrogen ion constituents in physiologic and pathologic contexts (extracellular pHs of 7.2–7.4 and 6.2–6.9, respectively). Both H^+^ and La^−^ are co-transported in an equimolar manner (1:1) across the cell membrane by monocarboxylate transporters. The authors provide pharmacologic evidence that MCT-1 is involved in this process. Given the important role of lactate as a fuel in other cell types, it is often challenging to explain how lactic acid selectively suppresses T cell function, independent of acidity. One possible scenario is that the accumulation of lactate impairs glycolysis by end-point inhibition, i.e., providing a negative feedback and causing a glycolytic shutdown. Consequently, an inability to recirculate cytoplasmic NAD^+^ and sustain glycolysis impairs ATP production, nucleotide biosynthesis and overall proliferation. Quinn and colleagues showed that exposure to high-lactate, low-glucose environments induced such reductive stress, culminating in decreased glycolysis and reduced serine production, in effector as well as regulatory T cells [40]. This may explain why (in the aforementioned study by Uhl and coworkers) the addition of oligomycin to the glycolytic stress test assay led to a decrease instead of an expected increase in ECAR in some assays. The cells may have an increased reliance on oxidative phosphorylation to support NAD^+^ production via the electron transport chain. However, rates of oxygen consumption (OCR) were also suppressed in lactic acid-treated T cells, so future work is necessary to explain these findings. Regardless, their data hints that sustained exposure to elevated levels of lactic acid above a critical threshold of 10 mM (which does not suppress metabolic function) and reaching 15 mM (led to metabolic crisis) suppresses T cell function.

As described above, T cell function was rescued by using sodium bicarbonate (NaBi) as a biological buffer [38]. How NaBi neutralises lactic acid in this scenario was not the focus of this paper. When T cells were preconditioned with lactic acid and NaBi for 48 h and then transferred to fresh Seahorse medium prior to analysis, there was no evidence of additional buffering during standard glycolytic stress test assays. These findings suggest that NaBi is not transported intracellularly. It is possible that 15 mM NaBi neutralises 15 mM lactic acid extracellularly, preventing its transport across the plasma membrane. Also interesting was that the addition of lactic acid and NaBi as conditioning factors led to superior glycolytic capacity in mouse T cells (a finding not observed in human T cells). How the experimental design influences this is unknown, but it is important to note that enhanced glycolytic capacity was measured after a 48-h conditioning regimen following a starvation period of up to 20 h in glucose free medium. This was then followed by exposure to RMPI medium (containing 11 mM glucose) and a further 15 mM glucose through the injection port. It is unlikely that these conditions giving rise to the metabolic phenotype would be encountered in any physiologic/pathologic context. These data were also generated using CD8+ T cells exclusively, and it would be interesting to see how AML-derived lactic acid influences CD4+ metabolic function or impacts the differentiation of CD4+ T cells to the regulatory (Tregs) or Th17 lineages. Although not considered in this study, NaBi can act as a precursor for important carboxylase reactions that are a part of key steps in metabolism [38].

As a buffering agent, NaBi activates carbonic anhydrase enzymes that convert acid into carbon dioxide and water. Clinically, NaBi has been used to treat tumor lysis syndrome [41]. It will be exciting to see if NaBi is used in conjunction with immunotherapy to boost T cell metabolic function in solid TMEs. Although speculative, NaBi may promote the expansion of T cells with enhanced quality during ex-vivo culture. Activated T cells undergo high rates of Warburg metabolism, and including 15 mM NaBi to medium formulations may give rise to T cell progeny with enhanced functional competence.

## 4. VISTA Is a pH-Sensitive Metabolic Checkpoint in the TME

Two interconnected features of the solid tumor environment, hypoxia and acidity, create an important barrier to adoptive immunotherapies. Hypoxia accentuates the reliance on glycolysis, culminating in lactic acid production, and the extrusion of protons into the extracellular space. Consequently, intratumoral pH levels can decline from 7.4 to 5.85 [42]. VISTA is a novel co-inhibitory ligand belonging to the B7 family of Immunoglobulins (Ig) [43]. VISTA is a homologue of PD-1, and also functions as an immune checkpoint molecule [44]. VISTA is highly expressed in tumor-infiltrating myeloid cells. Once activated, VISTA suppresses T cell proliferation and cytokine production. Structurally, VISTA contains a variable immunoglobulin domain containing several histidine residues in the extracellular chain [45]. At acidic pH, the imidazole ring becomes protonated, polar and hydrophilic. Johnston and coworkers developed an innovative screening assay to distinguish candidate receptors that bind to VISTA a pH-dependent manner [46]. They demonstrated that VISTA binds to PSGL-1, a glycoprotein that regulate extravasation from the blood to tissues (using selectins as their corresponding ligands) [46]. At low pH, histidine protonation promotes an interaction with negatively charged sulfated tyrosine, as well as glutamic acid residues found on PSGL-1. Such structural insights led to a model where VISTA acts as a pH-sensitive switch that dials down T cell activity [46]. Replacing three critical histidine residues of H153, H154, and H155 in the extracellular chain with negatively charged aspartic acid abrogated the interaction of VISTA with PSGL-1; moreover, substituting H98, H100, H153, H154 and H155 with a positively charged amino acid (arginine) restored VISTA:PSGL-1 binding, but pH selectivity was lost.

Several studies have implicated a role for VISTA in immune suppression in tumors. In prostate cancer, VISTA has emerged as an important factor underlying immune evasion following ipilimumab treatment [47]. In an adaptive response to ipilimumab treatment, macrophages induce VISTA forming an important immune inhibitory pathway within the prostate tumor microenvironment. Enhanced VISTA expression in hematopoietic cells that infiltrate metastatic melanomas is also correlated with disease severity and worsened prognosis [48]. Targeted inhibition of VISTA has emerged as an important strategy to overcome immune resistance and bolster the pre-existing immune response against cancer. Le Mercier and colleagues showed that disrupting VISTA:PSGL-1 interactions with monoclonal antibodies enhanced the anti-tumor function of adoptively transferred T cells in mouse models of colorectal adenocarcinoma and melanoma [44]. As the VISTA:PSGL-1 interaction is enhanced by the acidic TME, combination therapy with pH regulators and VISTA checkpoint monoclonal antibodies may have additive benefits for next-generation immunotherapies. Taken together, these studies identify a novel checkpoint inhibitor, induced in acidic, hypoxic tumors—further exemplifying the hostile soil within tumors that antigen-specific T cells must operate. Increasing evidence suggests that VISTA expression is permissive for the select expansion of tumor cells treated with checkpoint blockers. Future studies will likely reveal how the disrupted pH balance that facilitates the immune-inhibitory role of VISTA influences the hierarchy of substrate metabolism in this competitive microenvironment. Importantly, acidosis can impede other aspects of T cell function, including cytolytic activity [49].

## 5. Targeting MCTs to Remodel the TME

**Figure 1:**
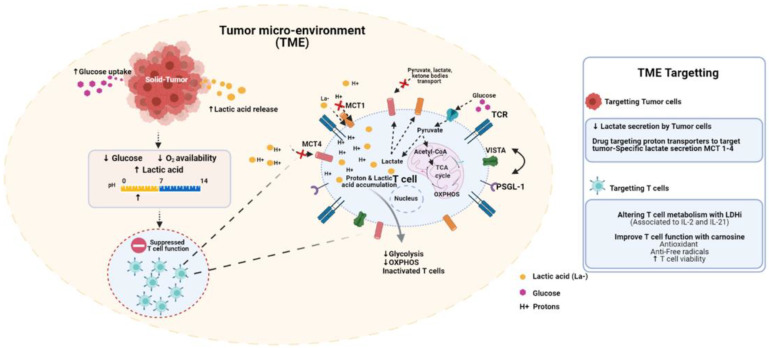
The tumor microenvironment (TME) is acidic and immune-suppressive. Tumor proliferation is distinguished by enhanced glucose consumption and lactic acid production. In response to increased tumor cell metabolism, the surrounding milieu becomes increasingly hypoglycemic, hypoxic and acidic. These conditions are inherently immune-suppressive. Low glucose and oxygen levels impair T cell metabolic activity. Acidosis inhibits glycolysis and downstream oxidative phosphorylation. Low pH induces V-domain Ig suppressor of T cell activation (VISTA), an immune-inhibitory checkpoint molecule. Several strategies have been developed to counter these effects. Nonsteroidal anti-inflammatories (NSAIDS), including diclofenac, can selectively inhibit MCT1-4 on cancer cells. Ex-vivo conditioning strategies, including the use of LDH inhibitors, can limit T cell terminal differentiation prior to adoptive transfer. Treating T cells with carnosine ex-vivo can shift their metabolic state from glycolytic/acidic to oxidative.

Immune and metabolic checkpoints can limit T cell effector function and persistence following adoptive transfer (Figure 1). Although therapies with immune checkpoint inhibitors offer a solution to restore function in exhausted T cells, such therapies have limited applicability and durability [50]. Targeting the immune-suppressive effects of acidity in the TME has attracted considerable interest, and warrants further discussion.

Strategies to limit lactate secretion by targeting MCT isoforms have been proposed as a potential adjuvant to T cell adoptive transfer. However, T cells share many metabolic features with tumor cells, including an induction of MCT4 following TCR engagement and CD28 co-stimulation, and thus compounds designed to inhibit MCT isoforms may prove antagonistic to T cell-mediated responses. Diclofenac is a monocarboxylic acid with known anti-inflammatory properties [51]. Diclofenac can also suppress tumor cell metabolism and proliferation by inhibiting MCT4 [51]. Remarkably, diclofenac has minimal impact on T cell cytokine production, as measured by the levels of IFN-γ and tumor necrosis factor (TNF) in vitro [52]. These findings position diclofenac as a potential therapeutic to selectively disable tumor cell metabolism in the TME. In T cell:tumor cell co-cultures, diclofenac enhanced T cell cytolytic responses. These findings highlight the potential benefit of targeting tumor-specific lactate secretion using diclofenac and its derivatives in the TME.

## 6. Altering T Cell Metabolism with LDHi

Interleukin-2 (IL-2) and Interleukin-21 (IL-21) are two closely related families of signaling molecules that arose from gene duplication. Both cytokines are pleiotropic molecules that support the maturation and differentiation of CD8+ effector T cells [53]. IL-2 has been widely used in adoptive immunotherapy as a soluble growth factor that supports the expansion of T cells ex-vivo, and the growth and differentiation of adoptively transferred tumor-reactive T cells in vivo [54]. However, IL-2 can promote the development of T cells into terminally differentiated effector cells, which limits durable efficacy following adoptive transfer. IL-2 can also trigger the development of regulatory T cells. IL-21 is an important factor that supports the development of Th17 cells, a subset with enhanced anti-tumor function [55]. Metabolically, IL-2 and IL-21 are diametrically opposed: IL-2 drives aerobic glycolysis in a PI3K-AKT-dependent manner while IL-21 activates STAT5 signal transduction; culminating in an oxidative rather than glycolytic state [56]. The metabolic phenotypes conferred by IL-2 and IL-21 have been proposed to be a key determinant underlying their differential antitumor activity.

In a recent study, Hermans and coworkers compared the metabolic state of IL-2 versus IL-21 primed T cells. Murine T cells were activated with dynabeads containing immobilised antibodies against CD3 and CD28 for two days, and then supplemented with either IL-2 or IL-21 for a further 48 h. Metabolically, IL-21 retained metabolic properties of quiescent T cells, as shown by extracellular flux analyses. In contrast, the energy cost to sustain IL-2 cultures was larger, exemplified by increased rates of basal and maximal oxygen consumption rates. Increased metabolic demands at rest, and following stimulation, led to a reduced overall energy generating capacity. A deficit in the contingency energy reserve may impede the persistence and anti-tumor function of adoptively transferred T cells, especially in hostile environments. Importantly, IL-2 and IL-21-treated cells show a difference in glucose consumption rates, as well as the expression of certain metabolites including lactate and pyruvate [57]. The lactate/pyruvate ratio, an important biomarker for glycolytic activity, was increased in IL-2 relative to IL-21 cultures. Attempts to revert IL-2-primed T cells to an oxidative state or accentuate substrate oxidation in IL-21-stimulated cultures relied on the use of a small molecule inhibitor against LDH.

LDH catalyses the reduction of pyruvate to lactic acid. *Ldh* mRNA expression is higher in IL-2 relative to IL-21-treated cultures. IL-2-treated cells secrete more lactate and protons, as measured by extracellular acidification rates in a Seahorse Assay. IL-2-treated cells also express more of the phosphorylated, inactive form of pyruvate dehydrogenase (PDH). PDH controls the metabolic fate of pyruvate, driving it towards cytoplasmic rather than mitochondrial metabolism [57]. This explains how IL-2 limits the oxidation of pyruvate for energy and TCA anaplerosis. LDHi resulted in a substantial decrease in lactate secretion and glucose consumption in both IL-2- and IL-21-treated cells [57]. In IL-2-treated cells, LDHi increased the intracellular pyruvate/lactate and NADH/NAD+ ratio significantly. Conceptually, increasing pyruvate availability by LDHi facilitates its entry and oxidation into the mitochondria by mass action. In their ^13^C_6_ glucose tracer assays, there was limited evidence that pyruvate metabolism in the TCA cycle was dramatically increased by LDHi. Follow-up studies are necessary to tease out the mechanisms by which LDHi treatment ex-vivo led to improved anti-tumor function following infusion, especially as the compound targets A and B isoforms of LDH. Comparatively, eliminating LDHA at the level of the germline gives rise to T cell progeny with limited anti-tumor function suggesting the temporal regulation of LDH activity is an essential consideration for adoptive immunotherapies. Overall, their promising data support a model in which Ldhi during ex-vivo culture confers unique metabolic benefits. In contrast, LDHA is indispensable to tumor-reactive T cells following adoptive transfer [57].

## 7. Carnosine Buffers TME and Improves T Cell Function

An important aspect of adoptive immunotherapy is the isolation and expansion of T lymphocytes ex-vivo to generate increased numbers of “sentinels” that recognise and target cancer cells following reinfusion. Understanding how medium formulations impact the effectiveness of T cell therapies is an exciting new area of research. Recently, we showed how a unique medium formulation, that was custom adjusted to mimic physiologic microenvironments, gave rise to CAR T-cell progeny with enhanced anti-tumor function in human xenograft models of neuroblastoma [58].

Using metabolomics, we screened a blood-based growth factor concentrate “Physiologix”, and identified increased levels of the biogenic amine carnosine relative to human serum. Carnosine is an L-histidine/beta-alanine dipeptide, abundantly found in skeletal muscle. It is well established that carnosine is an important physiochemical buffer to reduce muscle fatigue and lactic acid buildup during high-energy activities [59]. Carnosine is also an effective antioxidant and free-radical scavenger and has been shown to limit DNA damage and cellular stress [60]. These observed properties positioned carnosine as an excellent candidate to study in adoptive immunotherapy. Future efforts will likely determine how carnosine influences T cell viability and clonal integrity during extended cultures at normoxia and hypoxia.

Remarkably, we showed that increased carnosine availability increased lentiviral transduction; an effect accentuated at low MOIs using VSV-pseudotyped lentivirus. VSV-pseudotyped virus enter cells via the LDH receptor, which becomes embedded in clathrin-coated pits. Following endocytosis, there is a pH-dependent release/fusion of virus particles prior to nuclear import and reverse transcription. The specific mechanisms by which carnosine enhances lentiviral-mediated transduction is unknown, but its intracellular accumulation in T cells [58], and the unique pKa of L-histidine in its dipeptide form, likely plays an important role.

In Seahorse assays, carnosine treatment significantly decreased ECAR. The intrinsic ability of carnosine to neutralise an increase in proton load turn shifted the metabolic preference of T cells to oxidative phosphorylation. As extracellular acidosis is a feature of hypoxic tumor environments, as well as a barrier to effective immunotherapies, our findings highlight carnosine as a novel factor to enhance the quality of T cells expanded ex-vivo for adoptive immunotherapies.

## 8. Conclusions

Mitigating the immunosuppressive effects of the TME is necessary to improve the efficacy of cancer treatments. The TME is distinctly acidic due to high rates of glucose metabolism in cancer cells, culminating in excess lactic acid production and secretion. The corresponding decrease in extracellular pH inhibits glycolysis in T cells by negative feedback, induces reductive stress at hypoxia, and activates inhibitory immune checkpoint molecules such as VISTA. Overcoming these metabolic effects will require innovative strategies to selectively target the tumor cells themselves, thereby indirectly supporting T cell function, or using combinatorial approaches to addresses both factors synergistically. It is clear from the rigorous examination of the studies described above that regulating pH to restore T cell functional competence in hostile soils will enhance the therapeutic potential of adoptive immunotherapies against cancer.

## Data Availability

Not applicable.
